# Simultaneous Placement of Three Thin‐Delivery Multi‐Hole Self‐Expandable Metallic Stents for Malignant Hilar Biliary Obstruction Using a Large‐Channel Duodenoscope

**DOI:** 10.1002/deo2.70271

**Published:** 2026-01-06

**Authors:** Akinobu Koiwai, Morihisa Hirota, Tomohiro Oikawa, Kei Ishikawa, Chihiro Yunomura, Takuro Nakaya, Takehito Itoh, Yuki Miyashita, Nana Inomata, Kennichi Satoh

**Affiliations:** ^1^ Division of Gastroenterology Tohoku Medical and Pharmaceutical University Miyagi Japan

**Keywords:** biliary drainage, duodenoscope, ERCP, self‐expandable metal stent, side‐by‐side

## Abstract

Endoscopic biliary drainage for malignant hilar biliary obstruction is technically demanding especially when multiple ducts require drainage. Plastic inside stents are widely used because of their removability and ease of exchange, but multiple insertion is often hindered by stent interference. Recently, a multi‐hole self‐expandable metallic stent (MHSEMS) with a thin 5.9‐Fr delivery system and side‐hole design has been developed to facilitate multi‐duct drainage while maintaining communication between biliary branches. An 86‐year‐old woman presented with fever and vomiting. Laboratory data showed severe inflammation and mild cholestasis. Non‐contrast computed tomography and magnetic resonance cholangiopancreatography revealed Bismuth IV hilar obstruction. Emergency endoscopic retrograde cholangiopancreatography achieved drainage by placing two 5‐Fr double‐pigtail plastic stents into the right anterior and left ducts. After improvement, reintervention was performed using a new duodenoscope (ED‐840T; FUJIFILM, Tokyo, Japan) with a 4.5‐mm working channel. Cholangiography confirmed dilatation of three hepatic ducts. Three MHSEMSs were inserted simultaneously through their thin 5.9‐Fr delivery systems and deployed under fluoroscopic guidance. All stents expanded adequately, achieving effective drainage. This approach may represent an alternative to multi‐inside‐stent placement, overcoming the technical limitations of stent interference.

## 1 Introduction

Endoscopic biliary drainage is the preferred first‐line treatment for malignant biliary obstruction (MHBO). However, achieving adequate drainage in Bismuth type III‐IV strictures remains technically demanding. The choice between a plastic inside stent (IS) and a self‐expandable metallic stent (SEMS) depends on the patient's condition, expected survival, and procedural feasibility [1, 2].

ISs are widely used because they avoid the duodenobiliary reflex and can be easily replaced when occluded. However, when multiple ISs are required, stent‐stent interference often makes insertion and positioning difficult, especially in cases involving three or more intrahepatic ducts.

In contrast, SEMS−particularly covered types−provided longer patency and larger luminal diameter but are more challenging to place in the hilar region. Recently, a multi‐hole SEMS (MHSEMS, HANAROSTENT Biliary Multi Hole Benefit; Boston Scientific, MA, USA, Figure S1A) has been developed. This novel stent incorporates multiple small side holes in the covered membrane to maintain communication between biliary branches and prevent segmental isolation [3]. Its thin 5.9‐Fr delivery system also enhances deliverability through narrow strictures and allows for potential multi‐stent insertion.

Concurrently, a newly released duodenoscope (ED‐840T; FUJIFILM, Tokyo, Japan, Figure S1B) features a 4.5‐mm large working channel−the widest among current therapeutic scopes−enabling parallel device manipulation. Despite the larger channel, the outer diameter remains slender (13.1 mm), and no difficulties in reaching the papilla were encountered.

## 2 Case Report

**FIGURE 1:**
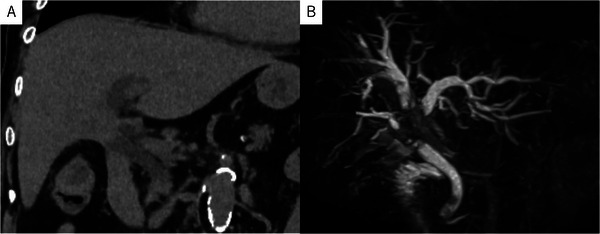
Non‐contrast CT showing wall thickening of the hilar bile duct and dilatation of the intrahepatic bile ducts. MRCP demonstrating dilated intrahepatic bile duct−right anterior, right posterior, and left hepatic ducts−with abrupt obstruction at the hepatic hilum. CT: computed tomography, MRCP: magnetic resonance cholangiopancreatography.

**FIGURE 2:**
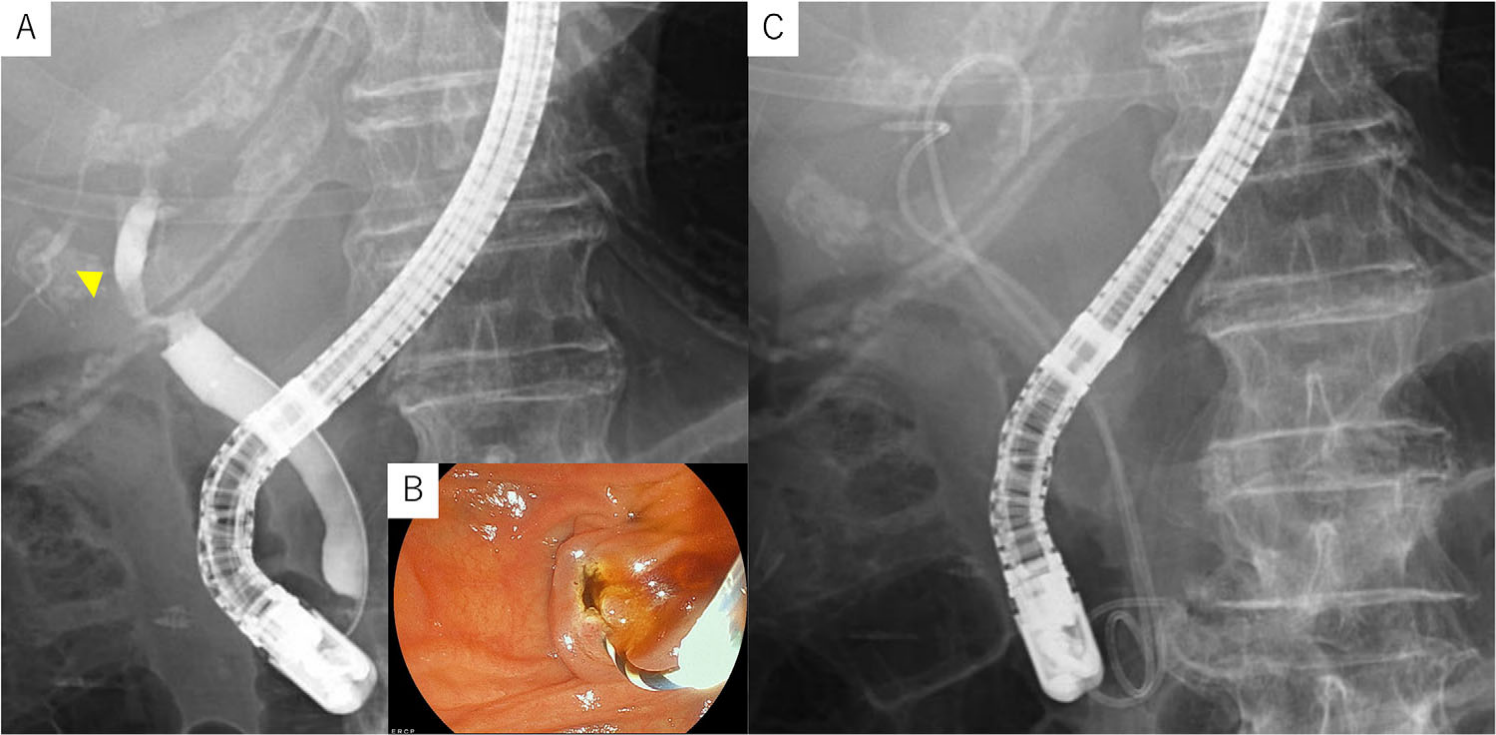
Cholangiography revealed a hilar biliary stricture. The dilated right posterior duct is indicated by a yellow arrowhead. Endoscopic image of the papilla after a small EST was performed. Placement of two 5‐Fr double‐pigtail plastic stents into the right anterior duct and the left hepatic duct for initial drainage. EST: endoscopic sphincterotomy.

An 86‐year‐old woman with hypertension and dyslipidemia presented with fever (39°C), vomiting, and poor appetite. Laboratory tests showed severe inflammation (WBC 15,400/µL, CRP 40.97 mg/dL, procalcitonin 73.34 ng/mL) and elevated hepatic and biliary enzymes. Computed tomography (CT) revealed intrahepatic bile duct dilatation and wall thickening at the hepatic hilum (Figure 1A). Magnetic resonance cholangiopancreatography (MRCP) demonstrated a Bismuth type IV hilar stricture (Figure 1B). Cytology obtained during this initial endoscopic retrograde cholangiopancreatography (ERCP) confirmed the diagnosis of hilar cholangiocarcinoma (Stage IIIB). Because of severe inflammation and advanced age, emergency ERCP was performed to achieve rapid decompression. Cholangiography revealed a high‐grade hilar stricture (Figure 2A). A small endoscopic sphincterotomy (EST) was performed (Figure 2B), and subsequently, two 5‐Fr double‐pigtail plastic stents (Biliary Stent, Medi‐Globe, Achenmuhle, Germany) were placed into the right posterior and left bile ducts (Figure 2C) as temporary drainage during the emergency procedure. The patient's condition improved rapidly following the procedure, accompanied by a steady decline in inflammatory markers and normalization of hepatic and biliary enzymes.

**FIGURE 3:**
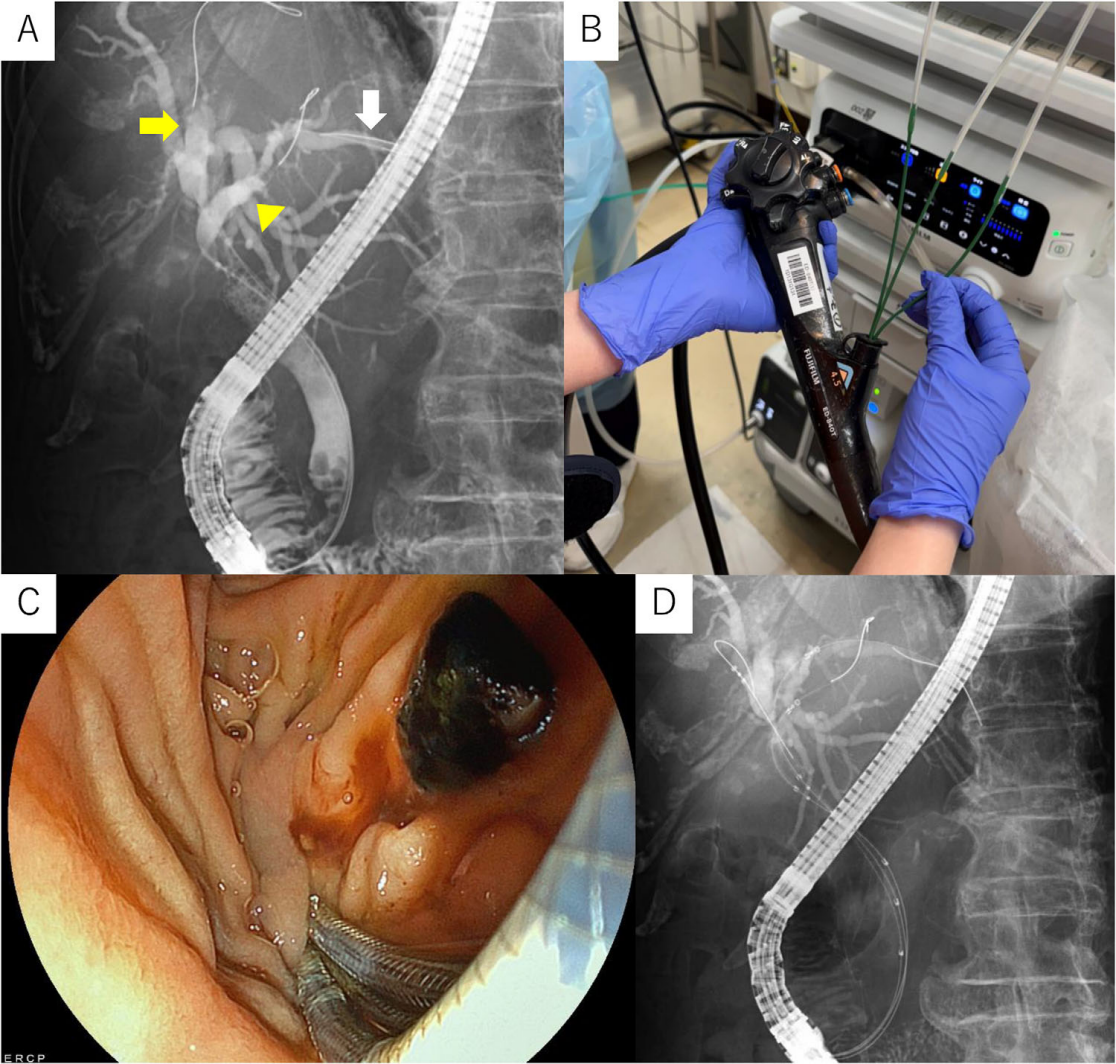
Guidewire selectively advanced into the right anterior (yellow arrow), right posterior (yellow arrowhead), and left hepatic ducts (white arrow). Under fluoroscopic guidance, three thin‐delivery MHSEMSs were inserted simultaneously over the guidewires and smoothly advanced into position. Endoscopic image during stent deployment. Fluoroscopic view confirming successful simultaneous insertion of the three MHSEMSs. MHSEMSs: multi‐hole self‐expandable metallic stents.

**FIGURE 4:**
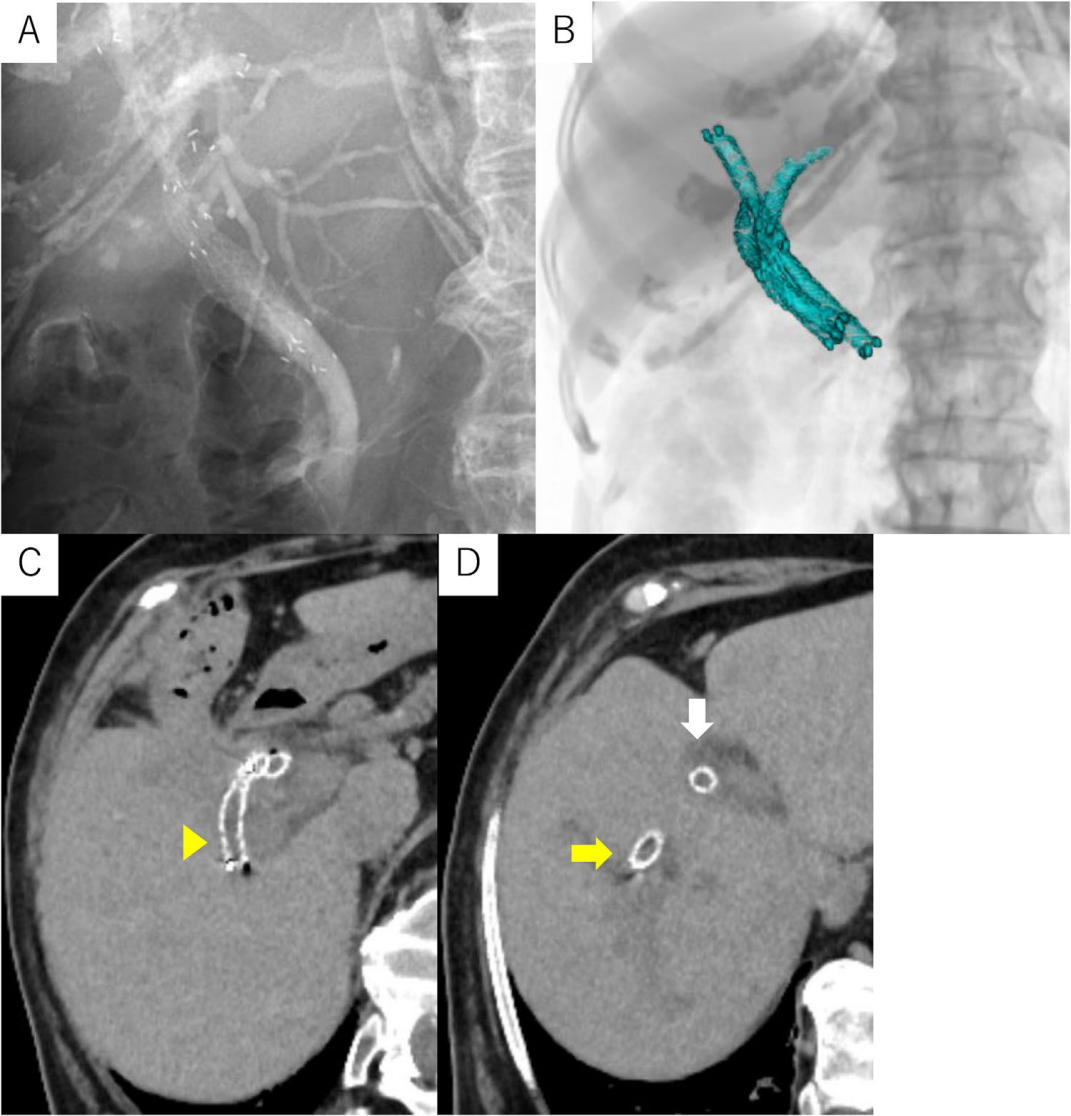
Fluoroscopic image after simultaneous deployment of three MHSEMSs. (A, B) Three‐dimensional reconstruction from post‐procedure CT demonstrating the configuration of deployed stents within the intrahepatic ducts. (C, D) Post‐procedural axial CT demonstrating correct placement of the three MHSEMSs in the right anterior (yellow arrow), right posterior (yellow arrowhead), and left hepatic ducts (white arrow). MHSEMSs: multi‐hole self‐expandable metallic stents, CT: computed tomography.

After clinical stabilization, definitive drainage was planned. The ED‐840T duodenoscope, equipped with a 4.5‐mm working channel, was used. Cholangiography showed mild residual dilatation of the right anterior, right posterior, and left ducts. The right posterior duct coursed unusually toward the left side, making selective guidewire insertion difficult. Before SEMS deployment, stone extraction was performed during the second ERCP, as a distal common bile duct (CBD) stone had been identified previously. Three 0.025‐inch guidewires were advanced selectively into each duct (right anterior: EndoSelector; Boston Scientific, MA, USA, right posterior: Fielder 25, ASAHI INTECC; Aichi, Japan, left: TRU wire; MEDICO'S HIRATA, Osaka, Japan) (Figure 3A). Because the large channel permitted simultaneous device insertion, three 5.9‐Fr MHSEMS delivery systems were introduced in parallel (Figure 3B,C). The stents (right anterior: 6 mm×8 cm; right posterior: 6 mm×6 cm; left: 6 mm×8 cm) were deployed simultaneously under fluoroscopy (Figures 3D and 4A) (Video S1). Both the operator and the assistant were trainees; the total procedure time from scope insertion to scope removal was 57 min, and the time required from completion of guidewire placement to the stent deployment was 6 min. A post‐procedural CT confirmed correct placement in all three ducts (Figure 4B–D). Given her advanced age, the best supportive care was chosen. During the 2‐month follow‐up, no recurrent biliary obstruction occurred.

## 3 Discussion

This case demonstrates the feasibility and clinical value of simultaneous placement of three thin‐delivery MHBO. Conventionally, multiple stents are placed sequentially, requiring repeated device exchanges and prolonged procedure time. These methods risk wire dislodgement and stent misalignment. To our knowledge, simultaneous deployment of three SEMSs has not been reported previously. The principal innovation was the combination of a large‐channel duodenoscope (ED‐840T) and a thin‐delivery system. Consequently, three stents were positioned and expanded simultaneously without interference, shortening the procedure time.

In daily practice, plastic ISs remain widely used because they are removable and reduce duodenobiliary reflux [1]. However, when multiple ISs are placed, the second and subsequent stents frequently face resistance at the hilar confluence, resulting in stent‐stent interference and occasional displacement of the initially placed stent. By deploying three MHSEMSs simultaneously, our method completely avoids this sequential interference and allows accurate positioning of all stents in a single step. Partial stent‐in‐stent placement using metallic stents is another established option; however, this method usually requires uncovered SEMSs, which cannot be removed once deployed. Consequently, re‐intervention becomes technically difficult if occlusion occurs. In contrast, MHSEMSs are covered and potentially removable, providing an important practical advantage for long‐term management. Although robust data on prolonged patency are lacking, the MHSEMS design has the potential to reduce the need for frequent re‐intervention compared with ISs. Given that malignant hilar obstruction often requires repeated interventions, the ability to achieve both multi‐duct drainage and future removability is a significant benefit of this technique.

The side‐hole design of the MHSEMS provides another key advantage. It maintains bile communication between intrahepatic segments while the covered membrane prevents tumor ingrowth, combining the strengths of ISs and covered SEMS. In the present case, all three stents expanded well and maintained drainage during follow‐up, without cholangitis or obstruction. However, the MHSEMS also has several potential disadvantages. Because the stent expands gradually after deployment, a slight positional shift may occur, which requires precise adjustment of the release point. In addition, the theoretical risk of overexpansion should be noted; placing three 6‐mm stents side‐by‐side may require a circumscribed diameter of approximately 13 mm, which could be problematic in patients with a narrow distal CBD. In the present case, no adverse events occurred, but the long‐term safety of this configuration remains uncertain. Regarding the existing literature, several reports have described triple metal stenting for MHBO, including combined side‐by‐side and stent‐in‐stent methods and the use of 6‐mm covered or uncovered SEMSs, with acceptable safety profiles when carefully selected patients are treated [4]. However, these data are still limited, and no firm consensus has been reached about the maximum acceptable number and size of stents in a narrow distal CBD. Another limitation concerns re‐intervention. Although MHSEMSs are covered and theoretically removable, there is no published evidence regarding the safety of removing three MHSEMSs placed side‐by‐side. At our institution, removal of two MHSEMSs above the papilla has been feasible using biopsy forceps without complications; however, long‐term removability after prolonged indwelling remains unclear. Careful patient selection is therefore essential.

Simultaneous triple MHSEMS deployment thus represents a new strategy for multi‐duct drainage in complex MHBO, offering both procedural efficiency and mechanical stability. Whether simultaneous or sequential deployment is superior is still unclear; simultaneous deployment allows fine adjustment of all stents while still constrained, whereas sequential deployment simplifies monitoring but limits positional correction once the first stent is released. The optimal approach requires further evaluation.

Cost considerations remain important. While three SEMSs impose a higher initial cost, they may reduce the need for repeated interventions, which can be advantageous in frail or elderly patients receiving best supportive care. Future cost‐effectiveness studies will be needed to define optimal case selection.

This approach may be particularly beneficial for patients in whom multiple ISs are difficult to insert due to stent interference. Prospective studies are warranted to evaluate long‐term patency, cost‐effectiveness, and broader clinical applicability. The combination of a thin 5.9Fr‐delivery system and 4.5‐mm working channel enabled efficient, stable, and interference‐free multi‐stent placement. Compared with conventional multi‐IS placement, this approach overcomes stent interference and achieves rapid, effective drainage. The MHSEMS, with its side‐hole structure, may provide a promising alternative for complex hilar strictures, merging the advantages of ISs and covered SEMS.

## Author Contributions

**Akinobu Koiwai**: prepared the first draft of the manuscript. **Akinobu Koiwai**, **Morihisa Hirota**, **Tomohiro Oikawa**, **Kei Ishikawa**, **Chihiro Yunomura**, **Takuro Nakaya**, **Takehito Itoh**, **Yuki Miyashita**, and **Nana Inomata**: managed the patient; **Morihisa Hirota** and **Kennichi Satoh**: revised the manuscript. All authors approved the final version of the manuscript.

## Conflicts of Interest

The authors declare no conflicts of interest.

## Funding

None.

## Supporting information




**Supporting Figure 1**: (A) Photograph of the fine‐delivery MHSEMS used in this case. (Courtesy of Boston Scientific).
(B) External view of the new duodenoscope ED‐840T equipped with a 4.5‐mm working channel. (Courtesy of FUJIFILM)
MHSEMS: multi‐hole self‐expandable metallic stent.

**Supporting video 1**: A streamlined technique for simultaneous triple MHSEMS placement using a large‐channel duodenoscope, demonstrating parallel advancement across the hilar stricture and controlled deployment in three intrahepatic ducts.
MHSEMS: multi‐hole self‐expandable metallic stent.
