# Supplementary figures and images for: Simultaneous Placement of Three Thin‐Delivery Multi‐Hole Self‐Expandable Metallic Stents for Malignant Hilar Biliary Obstruction Using a Large‐Channel Duodenoscope
Source: DEN Open. 2026 Jan 6;6(1):e70271. doi: 10.1002/deo2.70271 (PMC12772437; doi:10.1002/deo2.70271)

## Slide 1
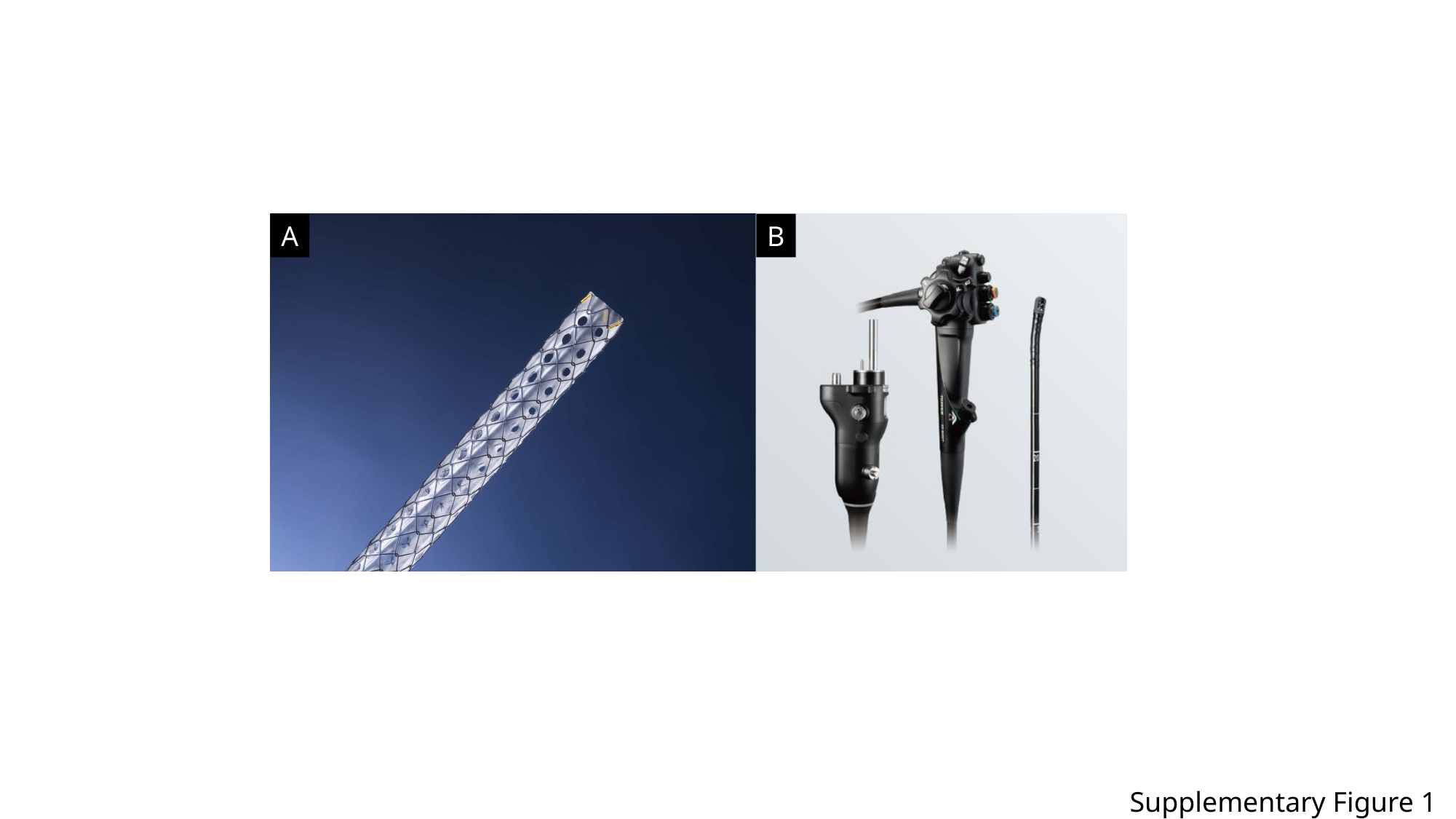

A
B
Supplementary Figure 1

Supplement: Supplementary file 1 — Supporting Figure 1: (A) Photograph of the fine‐delivery MHSEMS used in this case. (Courtesy of Boston Scientific). (B) External view of the new duodenoscope ED‐840T equipped with a 4.5‐mm working channel. (Courtesy of FUJIFILM) MHSEMS: multi‐hole self‐expandable metallic stent. [file DEO2-6-e70271-s002.pptx]
